# Mitochondrial DNA induces nucleus pulposus cell pyroptosis via the TLR9-NF-κB-NLRP3 axis

**DOI:** 10.1186/s12967-023-04266-5

**Published:** 2023-06-15

**Authors:** Peng Lu, Huayong Zheng, Hao Meng, Chuan Liu, Lianhong Duan, Jianzheng Zhang, Zhicheng Zhang, Jie Gao, Yang Zhang, Tiansheng Sun

**Affiliations:** 1 Chinese PLA Medical School, Beijing, China; 2 Department of Orthopedics, Chinese PLA General Hospital, Beijing, China

**Keywords:** Intervertebral disc degeneration, Nucleus pulposus cells, mtDNA, TLR9, NF-κB, NLRP3 inflammasome, Pyroptosis

## Abstract

**Background:**

Nucleus pulposus cell (NPC) death and progressive reduction play important roles in intervertebral disc degeneration (IVDD). As part of a damage-associated molecular pattern, mitochondrial DNA (mtDNA) can be recognized by TLR9 and triggers the expression of NF-κB and NLRP3 inflammasomes, inducing pyroptosis and inflammatory response. However, whether mtDNA induces NPC pyroptosis via the TLR9-NF-κB-NLRP3 axis and promotes IVDD remains uncertain.

**Methods:**

We constructed an in vitro NPC oxidative stress injury model to clarify the mechanism of mtDNA release, TLR9-NF-κB signaling pathway activation, and NPC injury. We further verified the mechanism of action underlying the inhibition of mtDNA release or TLR9 activation in NPC injury in vitro. We then constructed a rat punctured IVDD model to understand the mechanism inhibiting mtDNA release and TLR9 activation in IVDD.

**Results:**

We used human NP specimen assays to show that the expression levels of TLR9, NF-κB, and NLRP3 inflammasomes correlated with the degree of IVDD. We demonstrated that mtDNA mediated TLR9-NF-κB-NLRP3 axis activation in oxidative stress-induced human NPC pyroptosis in vitro. Oxidative stress can damage the mitochondria of NPCs, causing the opening of the mitochondrial permeability transition pores (mPTP) and leading to the release of mtDNA into the cytosol. Furthermore, inhibition of mPTP opening or TLR9 activation blocked TLR9-NF-κB-NLRP3 axis activation and thereby mediated NPC pyroptosis and IVDD.

**Conclusion:**

mtDNA plays a key role in mediating NPC pyroptosis and IVDD via the TLR9-NF-κB-NLRP3 axis. Our findings provide new potential targets for IVDD.

**Supplementary Information:**

The online version contains supplementary material available at 10.1186/s12967-023-04266-5.

## Highlights

- NPC pyroptosis mechanism via mtDNA mediated TLR9-NF-κB-NLRP3 axis activation is unclear.
- We demonstrate that TLR9, NF-κB, and NLRP3 inflammasomes correlate with IVDD.
- mtDNA via the TLR9-NF-κB-NLRP3 axis mediates NPC pyroptosis and IVDD.

**Supplementary Information:**

The online version contains supplementary material available at 10.1186/s12967-023-04266-5.

## Background

Low back pain (LBP) is a syndrome that includes pain in the lower back, lumbosacral region, and buttocks, with a high incidence and disability rate [1, 2]. Intervertebral disc degeneration (IVDD) is considered the main cause of chronic LBP [3]. Current strategies for the management and treatment of IVDD focus on the relief of the corresponding symptoms, such as pharmacological pain relief and surgical resection; however, there are currently no measures that can mitigate disc degeneration over time [4]. Therefore, studying the molecular mechanisms of IVDD will help develop relevant strategies in the future. The nucleus pulposus (NP) plays an important role in buffering stresses between vertebral bodies. NP cells (NPCs) play a critical role in maintaining the balance of extracellular matrix (ECM) metabolism and physiological functions of the NP [5]. Studies have demonstrated that the IVDD process involves three interrelated events, all of which form a vicious circle: impairment of the biological function of NPCs, imbalance of ECM metabolic homeostasis, and changes in the internal environment of NP [6, 7]. The progressive loss of NPCs may be one of the initial events in IVDD. Non-programmed necrosis of NPCs can cause the expression of inflammatory molecules, such as NLRP3 inflammasomes and IL-1β, which further exacerbates the loss of NPCs and the imbalance of ECM metabolism, leading to a reduction in the biological function of NP and ultimately exacerbation of IVDD and LBP [8]. Therefore, to understand the molecular mechanisms underlying the abnormal loss of NPCs may provide new therapeutic targets for IVDD.

Oxidative stress (OS) can damage mitochondria, resulting in a decrease in mitochondrial membrane potential (MMP) and the opening of mitochondrial permeability transition pores (mPTP), allowing mitochondrial DNA (mtDNA) to be released into the cytosol [8-10]. mtDNA is a circular DNA fragment containing unmethylated CpG, a structure that can be recognized by pattern recognition receptors and induces damage-associated molecular pattern (DAMP) activation. As a kind of pattern recognition receptor, TLR9 (Toll-like receptor 9) is localized in the cytosol and specifically recognizes CpG fragments. Due to the presence of CpG fragments in mtDNA, mtDNA can be recognized by TLR9. Studies have reported that TLR9 recognized mtDNA and then triggered (nuclear factor-kappa B) NF-κB upregulation via MyD88, leading to NLRP3 and pro-IL-1β expression [11-13].

When cells sense injury signals, NLRP3 interacts with the pyrin domain of apoptosis-associated speck-like protein (ASC) containing a caspase recruitment domain to form a complex, after which pro-caspase-1 is recruited by the caspase recruitment domain of ASC, eventually polymerizes into the NLRP3 inflammasome [14]. The activated NLRP3 inflammasome acts as a platform to cleave pro-caspase-1 into caspase-1; caspase-1, in turn, cleaves pro-IL-1β into IL-1β and also cleaves gasdermin D (GSDMD), releasing the N-terminal domain of GSDMD, and the latter punches holes in the cell membrane, allowing IL-1β to be released extracellularly, inducing inflammation and pyroptosis [15, 16].

TLR9-NF-κB-NLRP3 axis activation is involved in various diseases, such as Alzheimer’s disease, nonalcoholic steatohepatitis, myocarditis, and other inflammatory diseases [12, 17-19]. Few studies have examined the contribution of TLR9-NF-κB-NLRP3 axis activation to IVDD. However, it has now been shown that overexpression of NLRP3 inflammasome and IL-1β is an important cause of IVDD [20, 21]. Therefore, we hypothesized that mtDNA-mediated TLR9-NF-κB-NLRP3 axis activation may play a significant role in IVDD, and blocking mtDNA-mediated TLR9-NF-κB-NLRP3 axis activation may alleviate IVDD development. Therefore, the aim of this study was to explore the role of mtDNA-mediated TLR9-NF-κB-NLRP3 axis activation in NPCs. We also investigated the effect of blocking mtDNA-mediated TLR9-NF-κB-NLRP3 axis activation in IVDD using a rat model.

## Materials and methods

### Ethics statement

The collection of medical records and imaging data, use of NP specimens, and experimentation on animals were performed according to the guidelines of the Ethics Committee of the PLA General Hospital and the “Guidelines for the Care and Treatment of Laboratory Animals” published by the National Institutes of Health. In accordance with the guidelines of the Declaration of Helsinki, the Ethics Committee of the PLA General Hospital approved the protocols (No. S2021-062-35).

### Collection of human NP specimens

We collected NP specimens from volunteers who underwent spinal surgery for spinal trauma, idiopathic scoliosis, or disc herniation, and who had no history of spinal infection, immunosuppression, hypertension, diabetes, or smoking. NP specimens were collected from 20 volunteers: 11 males and 9 females, aged 17–65 years. The degree of IVDD was evaluated by the Pfirrmann grading system. NP specimens from volunteers with spinal trauma or idiopathic scoliosis were both considered for the extraction of healthy NPCs in humans. We defined five samples from volunteers with idiopathic scoliosis as grade I and 15 samples from different segments of lumbar disc herniation as grades II–IV. Information pertaining to each participant is listed in Additional file 1: Table S1.

### Human NPC culture and treatment

Human NPCs were partially isolated from grade I samples, as described previously [20, 22]. Briefly, the collected NP specimens were cleaned with a sterile isotonic sodium chloride solution, cut into pieces, placed in a centrifuge tube filled with sterile isotonic sodium chloride solution, and transported quickly to the laboratory. NP specimens were digested with 0.25% type II collagenase (C8000, Solarbio) at 37 °C for 2 h. The obtained NPCs were cultured in an incubator with 5% CO_2_ at 37 °C. The cell culture medium consisted of DMEM/F12 (11320033, Gibco), 10% FBS (10100147, Gibco), and 1% penicillin/streptomycin (G4003, Servicebio). The culture medium was replaced every 3 days, and the second-passage cells were used for further in vitro experiments.

The OS injury model of NPCs was established in vitro by treating NPCs in culture medium with 100 µM or 200 µM H_2_O_2_ for 24 h. The control group was not treated with H_2_O_2_. To explore the effect of the inhibition of TLR9 activation in NPCs under OS, NPCs were treated with 5 µM E6446 (T4206, TargetMol) and 100 µM H_2_O_2_ for 24 h. To explore the effect of blocking mPTP from opening in NPCs under OS, NPCs were administered with 100 µM H_2_O_2_ and 10 µM cyclosporine (CsA) (59865-13-3, Sigma-Aldrich) for 24 h. E6446 is a specific TLR9 antagonist and inhibits DNA-TLR9 interaction [23]. CsA shows its effect on mitochondria by blocking mPTP opening [24].

### Western blotting

Following a previously used method [25], proteins derived from NPCs were obtained using RIPA (G2002, Servicebio) with 1.5% phosphatase inhibitor (G2007, Servicebio), electrophoretically separated through 8–20% SDS-PAGE, and blocked using 5% nonfat milk. Cell lysates were first incubated with a primary antibody and then a secondary antibody. Antibody information is listed in Additional file 1: Table S2. Western blotting reagents are listed in Additional file 1: Table S3. Protein bands were detected through chemiluminescent immunoassay, and GAPDH was used for normalization. The blots were imaged using the CLINX ChemiSciope 6100 imaging system. Image analysis was performed using the ImageJ software.

### Histological evaluation

For human NP specimens, paraffin blocks were cut into 4 μm sections. Hematoxylin and eosin (H&E) staining to assess the histological degeneration of IVDD. Additionally, for immunohistochemical analysis, sections were labeled with primary antibodies overnight at 4 °C. Sections were incubated with secondary antibodies for 1 h at 21 °C.

### Tyramide signal amplification for immunohistochemical enhancement

To assess the levels of TLR9 and NF-κB in human NP specimens, the sections were stained using fluorescent multiplex immunohistochemistry [26]. The sections were dewaxed and rehydrated with xylene and gradient ethanol, then subjected to antigen repair with pepsin for 30 min at 37 °C, followed by serum blocking for 30 min at 21 °C, and incubation with primary overnight at 4 °C and secondary antibodies for 50 min at 21 °C. Antibodies were coupled with tyramide-conjugated fluorophores as follows: TLR9 with iF488-Tyramide (G1231, Servicebio) and NF-κB with iF555-Tyramide (G1233, Servicebio). The immunolabeled sections were imaged using a fluorescence microscope (Eclipse 80i, Nikon).

### RNA interference

To knockdown TLR9, we transfected NPCs for 24 h using Lipofectamine 8000 (C0533FT, Beyotime) with 100 pmol TLR9 small-interfering RNA (siRNA) (Thermo Fisher Scientific). The experimental and control groups were immediately administered with 100 µM H_2_O_2_ for 24 h. The sequences of siRNA are listed in Additional file 1: Table S4.

### PCR

NPCs were treated with TRIzol (15596026, Invitrogen) to obtain total RNA and reverse-transcribed to generate cDNA using the SweScript RT I First Strand cDNA Synthesis Kit (G3330, Servicebio). DNA abundance was visualized using agar gel electrophoresis. RT-qPCR was performed for quantitative analysis of DNA content. The expression level was normalized to that of GAPDH. The PCR primers are listed in Additional file 1: Table S4.

### Cytosolic mtDNA assay

Cytosolic DNA and total DNA of NPCs were extracted using the NE-PER™ Nuclear and Cytosolic Extraction Reagents kit (78833, Thermo Fisher Scientific) following the manufacturer’s instructions. mtDNA abundance was visualized through agarose gel electrophoresis. RT-qPCR was used for quantitative analysis of mtDNA content. The expression level was normalized to that of GAPDH. The PCR primers are listed in Additional file 1: Table S4.

### ROS measurement

Cytosolic ROS were measured using H2DCFDA (D399, Invitrogen), and mitochondrial ROS were measured using MitoSOX Red mitochondrial superoxide indicator (M36008, Invitrogen). After the in vitro experiments, the samples were stained and detected following the manufacturer’s instructions.

### Mitochondrial quality, MMP, and mPTP opening assay

Mitochondrial quality was assessed using the Mito-Tracker Red CMXRos Assay Kit (M7512, Invitrogen). MMP was detected using the MitoProbe™ JC-1 Assay Kit (M34152, Invitrogen). mPTP opening was detected using the Mitochondrial Permeability Transition Pore Assay Kit (C2009S, Beyotime). All operations were performed following the manufacturers’ instructions.

### NPC viability assay

First, 2 × 10^6^ NPCs were plated onto 6-well cell plates, and after 48 h, the cells completely adhered. Then, cells were treated with different intervention factors (H_2_O_2_, CsA, or E6446) for 24 h. The NPCs were gently washed three times with PBS (G4202, Servicebio) and incubated with Calcein AM/PI (C2012, Beyotime) at 21 °C for 30 min. Images were obtained through fluorescence microscopy (Eclipse 80i, Nikon).

### IL-1β ELISA

After treatment with different intervention factors, 100 µl supernatant was collected from the NPC lysates. The expression level of human IL-1β was detected using the IL-1 beta Human ELISA Kit (KHC0011, Invitrogen) following the manufacturer’s instructions.

### Mitochondrial DNA-TLR9 interaction assay

After treatment with different intervention factors, the NPCs were lysed at 4 °C using Triton X-100 (85111, Thermo Fisher Scientific). The supernatant was collected after centrifugation. The supernatant was incubated with IgG isotype control monoclonal antibodies or anti-double-stranded DNA (dsDNA) at 4 °C for 8 h. Next, protein A/G magnetic beads (88802, Thermo Fisher Scientific) were added and co-incubated at 4 °C for 2 h. After incubation, beads were washed and separated into two portions. One portion was eluted by SDS buffer (P0013G, Beyotime), boiled at 95 °C for 10 min, and then analyzed by western blotting. The other portion of the beads was eluted by a chromatin immunoprecipitation elution buffer. After treatment with protease K, the TIANamp Genomic DNA Kit (4992254, Tiangen) was used to collect DNA, and PCR analysis was performed [27].

### Proximity ligation assay (PLA)

After treatment with different intervention factors, 4% paraformaldehyde fixation of the NPCs was performed at 21 °C for 10 min. The Duolink® In situ PLA® Kit (DUO94104, Sigma-Aldrich) was used to detect the physical binding of dsDNA with TLR9. All operations followed the manufacturer’s instructions.

### Transmission electron microscopy (TEM) observation of cellular microstructure

After treatment with different intervention factors, the NPCs were fixed with 2.5% glutaraldehyde (P1126, Solarbio) for 10 min and 1% osmium tetroxide (201030, Sigma-Aldrich) for 2 h at 21 °C. After washing with PBS, the samples were sequentially dehydrated, permeabilized, embedded, polymerized, and cut into sections of 60–80 nm. The tissues were filtered through 150-mesh cuprum grids with formvar film. Staining sections with uranium acetate and lead citrate, and then dried overnight. Images were obtained using TEM (HT7800, Hitachi, Japan).

### Animals and treatment

Sprague-Dawley rats (weight 200–250 g) were weighed, and pentobarbital 2% (w/v) was injected into the peritoneum (40 mg/kg). As described previously [20, 28], X-rays were used to position the Co6/7, Co7/8, and Co8/9 discs, which were punctured with a 27 G needle and held for 1 min. The pretest confirmed that the puncture depth was approximately 3–4 mm. CsA (10 µM) or E6446 (10 µM) was injected into the Co8/9 disc immediately after puncture, whereas an equal volume of PBS was injected after puncturing the Co7/8 disc. CsA was used to assess the role of the cytosolic mtDNA in IVDD. E6446 was used to assess the effect of TLR9 activation on IVDD. To avoid individual differences, we selected three neighboring intervertebral discs in the tail vertebrae of the same rat. We defined unpunctured Co6/7 discs as the negative control group (n = 5), punctured Co7/8 discs + PBS, and punctured Co8/9 discs + CsA or + E6446 as the experimental groups (n = 5).

### Imaging evaluation

One month after acupuncture, all the rats were scanned using X-ray and magnetic MRI. In all rats, 9.4 T animal MRI (BioSpec70/20USR, Bruker, Germany) was used to assess the signal characteristics in T2-weighted images. The rats were maintained prone with their tails in a straight line after anesthesia. As described previously, the disc height index was determined based on X-ray imaging, and the degree of IVDD was determined by the Pfirrmann grade. The imaging parameters are listed in Additional file 1: Table S5.

### Histopathologic analysis and immunohistochemical examination of rat model

After imaging, the rats were euthanized by peritoneal injection of 2% pentobarbital. Disc samples were formaldehyde-fixed, dehydrated, and paraffin-embedded. The disc samples were cut into sections of 4 μm. Subsequently, staining sections with H&E, Safranin O-fast green, and Masson’s trichrome. IVDD’s histological alterations were identified utilizing histopathologic findings. TLR9, NF-κB, and NLRP3 expression levels were measured using immunofluorescence staining.

### Statistical analysis

Data are presented as the mean ± SD. GraphPad Prism 9.0 (GraphPad Software, USA) was used to draw graphs and for statistical analysis. Two groups were compared using the Student’s t-test, and three groups were compared using one-way ANOVA and Tukey’s test.

## Results

### Levels of TLR9, NF-κB, and NLRP3 inflammasomes were associated with the degree of IVDD

**Fig. 1:**
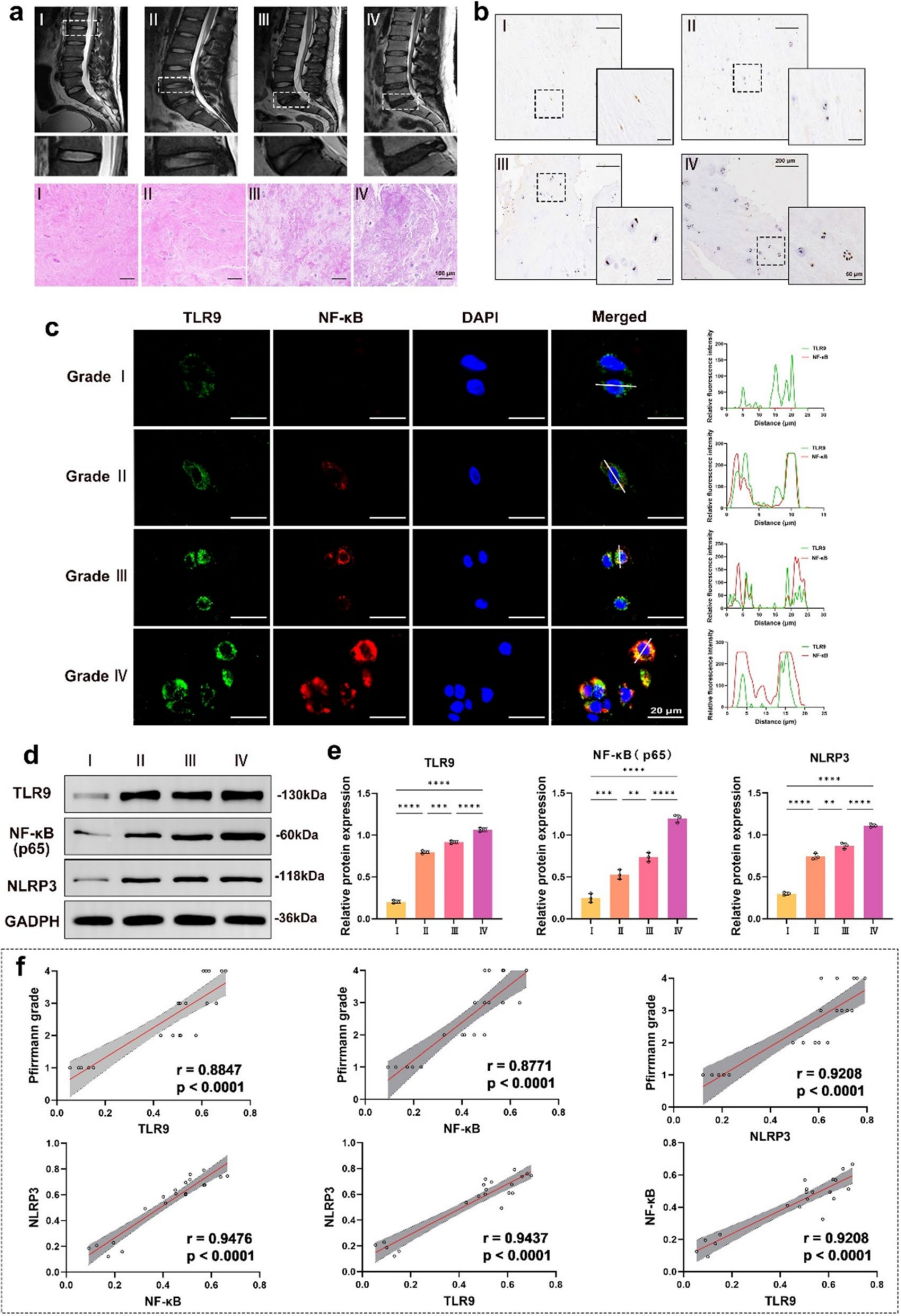
Levels of TLR9, NF-κB, and NLRP3 inflammasomes were associated with the degree of IVDD. **a** MRI and H&E images of NP with different degrees of IVDD. **b** Immunohistochemistry images of NLRP3 in NP with different degrees of IVDD. **c** TSA-IF images of TLR9 and NF-κB in NP with different degrees of IVDD. **d** Western blotting images of TLR9, NF-κB, and NLRP3 in NP with different degrees of IVDD. NP specimens for each grade of IVDD were obtained from 5 patients (n = 5), and each specimen was repeated for 3 times. **e** The quantitative analysis of TLR9, NF-κB, and NLRP3 expression levels in NP with different degrees of IVDD (n = 5). **f** Correlation analysis between the TLR9, NF-κB, NLRP3, and the Pfirrmann grades (n = 20). *p < 0.05, **p < 0.01, ***p < 0.001, ****p < 0.0001

To detect the levels of TLR9, NF-κB, and NLRP3 inflammasomes, we collected human NP specimens with different degrees of IVDD. MRI T2-weighted images showed different signal intensities in healthy and degenerated NP (Fig. 1a). The results showed that the higher the degree of IVDD, the lower the water content of NP. In addition, NP with higher degrees of degeneration shrinks in volume compared with NP with lower degrees of degeneration, which subsequently leads to a decrease in disc height. H&E staining showed structural disorganization and reduced ECM in degenerated NP tissue, especially in grades III and IV (Fig. 1a). Immunohistochemistry showed that the higher the degree of IVDD, the higher the level of NLRP3 inflammasomes (Fig. 1b). Tyramine signal amplification immunofluorescence (TSA-IF) showed enhanced fluorescence signal of TLR9 and NF-κB with increasing degeneration of IVDD (Fig. 1c). Western blotting showed that the TLR9, NF-κB, and NLRP3 inflammasome expression was increased in IVDD (Fig. 1d, e). Linear regression analysis revealed a positive correlation between TLR9, NF-κB, and NLRP3 inflammasome expression levels and the degree of IVDD (Fig. 1f). We also investigated the association between the TLR9, NF-κB, and NLRP3 inflammasomes, and discovered a substantial positive correlation. The above results suggest that the TLR9-NF-κB-NLRP3 axis activation is associated with IVDD.

### OS induced activation of the TLR9-NF-κB-NLRP3 axis and NPC pyroptosis

**Fig. 2:**
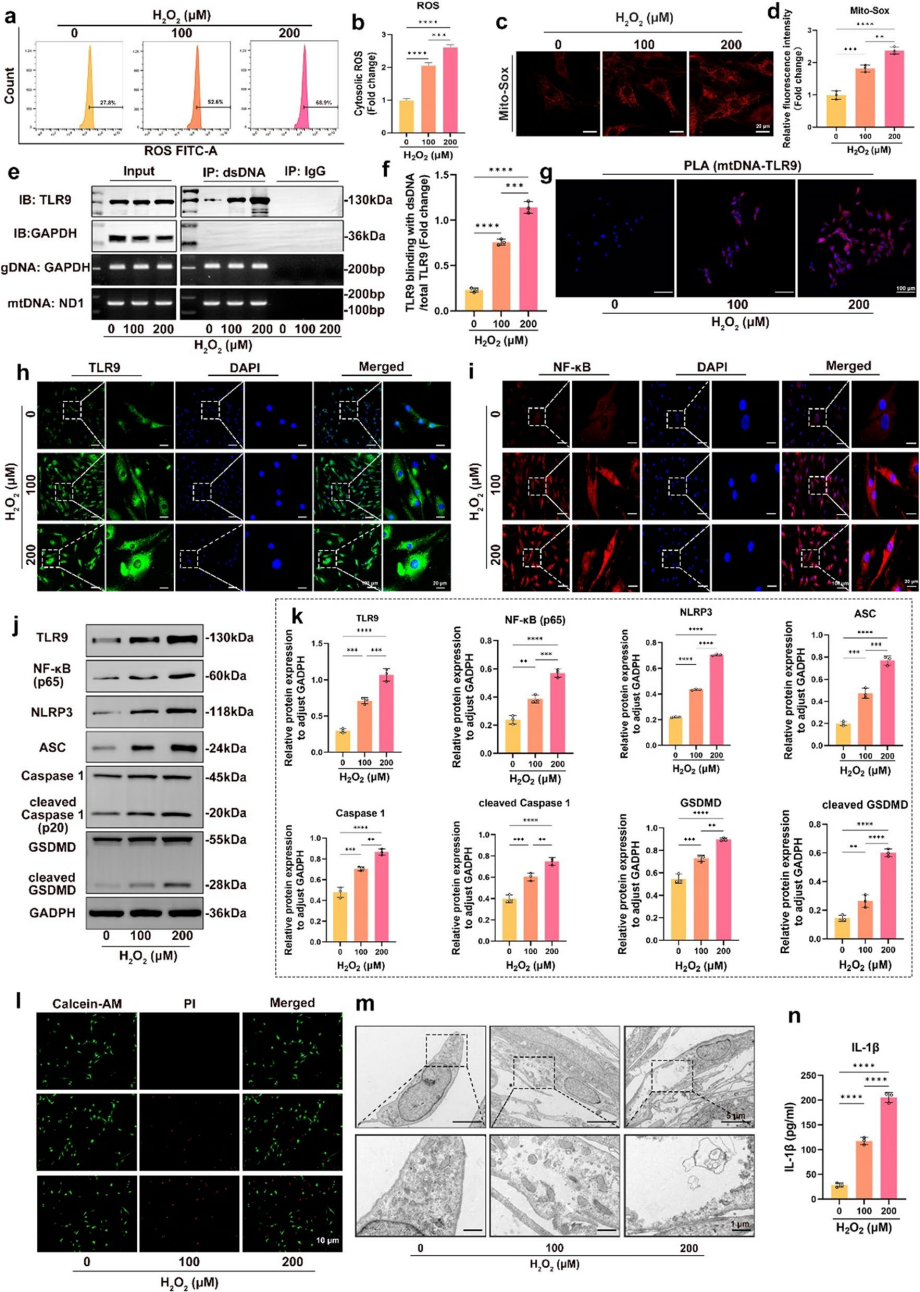
OS induced activation of the TLR9-NF-κB-NLRP3 axis and NPC pyroptosis. Human NPCs was cultured with 0, 100, and 200 µM H_2_O_2_ for 24 h. **a** Scatter plots of H2DCFDA staining for cytoplasmic ROS. **b** Quantitative analysis of H2DCFDA staining for cytoplasmic ROS. **c** Fluorescence images of MitoSOX for mitochondrial ROS. **d** Quantitative analysis of fluorescence intensity of MitoSOX for mitochondria ROS. **e** Immunoprecipitation images of mtDNA-TLR9 interaction. **f** Quantitative analysis of the level of mtDNA-TLR9 interaction. **g** The PLA immunofluorescence images of mtDNA-TLR9 interaction. **h** Immunofluorescence images of TLR9 in NPCs. **i** Immunofluorescence images of NF-κB in NPCs. **j** Western blotting images of TLR9, NF-κB, NLRP3, ASC, pro caspase-1, cleaved caspase-1, GSDMD, and cleaved GSDMD in NPCs. **k** Quantitative analysis of the expression levels of TLR9, NF-κB, NLRP3, ASC, pro caspase-1, cleaved caspase-1, GSDMD, and cleaved GSDMD in NPCs. **l** Fluorescence images of Calcein-AM/PI staining in NPCs. **m** TEM images of the NPCs. **n** Quantitative analysis of IL-1β in NPCs by ELISA. *p < 0.05, **p < 0.01, ***p < 0.001, ****p < 0.0001

Recent research has shown that OS may cause inflammatory damage in NP, resulting in the phenotype conversion of NPCs and the development of IVDD [29, 30]. To further explore the mechanism of NPC damage, NPCs were treated with different levels of H_2_O_2_ for 24 h. Subsequently, H2DCFDA and MitoSOX were used to measure the levels of cytosolic and mitochondrial ROS, respectively. A dose-dependent increase in the ROS levels in the cytosol and mitochondria were observed in the NPCs after 24 h of culturing with H_2_O_2_ (Fig. 2a–d). Moreover, to clarify whether mtDNA-TLR9 interaction was increased in NPCs under OS, we performed western blotting to assess the DNA-TLR9 interaction, which indicated a substantial enrichment of TLR9 levels in the H_2_O_2_-treated group (Fig. 2e, f). We performed PLA to clarify mtDNA-TLR9 interaction in NPCs, and the results showed that the level of mtDNA-TLR9 interaction in the H_2_O_2_-treated groups were substantially increased (Fig. 2g and Additional file 2: Fig. S2a).

To clarify whether OS activated the TLR9-NF-κB axis in NPCs, immunofluorescence staining and western blotting were performed. Immunofluorescence images showed a dose-dependent increase in the levels of TLR9 and NF-κB in NPCs after culturing with H_2_O_2_ for 24 h (Fig. 2h, i and Additional file 2: Fig. S1a). The western blotting results showed a dose-dependent increase in the levels of TLR9 and NF-κB in NPCs after culturing with H_2_O_2_ for 24 h (Fig. 2j, k). These data suggest that TLR9 recognizes and binds to mtDNA and then activates NF-κB in NPCs under OS. OS leads to NLRP3 inflammasome activation, which subsequently mediates GSDMD activation via caspase-1 [31]. GSDMD punches the cytomembrane, leading to IL-1β release and pyroptosis. To determine whether OS mediates NPC pyroptosis via the NLRP3 inflammasomes, we treated NPCs with different doses of H_2_O_2_ and subsequently examined the expression levels of relevant proteins through western blotting. The results showed that the levels of NLRP3, ASC, caspase-1, cleaved caspase-1, GSDMD, and cleaved GSDMD increased in NPCs with increasing doses of H_2_O_2_ (Fig. 2j, k). In addition, ELISA results showed increased expression of IL-1β in the H_2_O_2_-treated group, indicating that OS promoted the expression of inflammatory factors in NPCs (Fig. 2n). We used Calcein-AM and PI staining to assess cell viability and showed that NPC pyroptosis increased substantially with increasing doses of H_2_O_2_ (Fig. 2l). The ultra-microstructure of NPCs was observed using TEM, and substantial cell swelling was found in the NPCs of the H_2_O_2_-treated groups, which is a characteristic manifestation of GSDMD punching the cytolemma and leads to pyroptosis (Fig. 2m). The above data indicated that OS induced NPC pyroptosis via the TLR9-NF-κB-NLRP3 axis.

### Knockdown or inhibition of TLR9 activation reduced NPC pyroptosis under OS

**Fig. 3:**
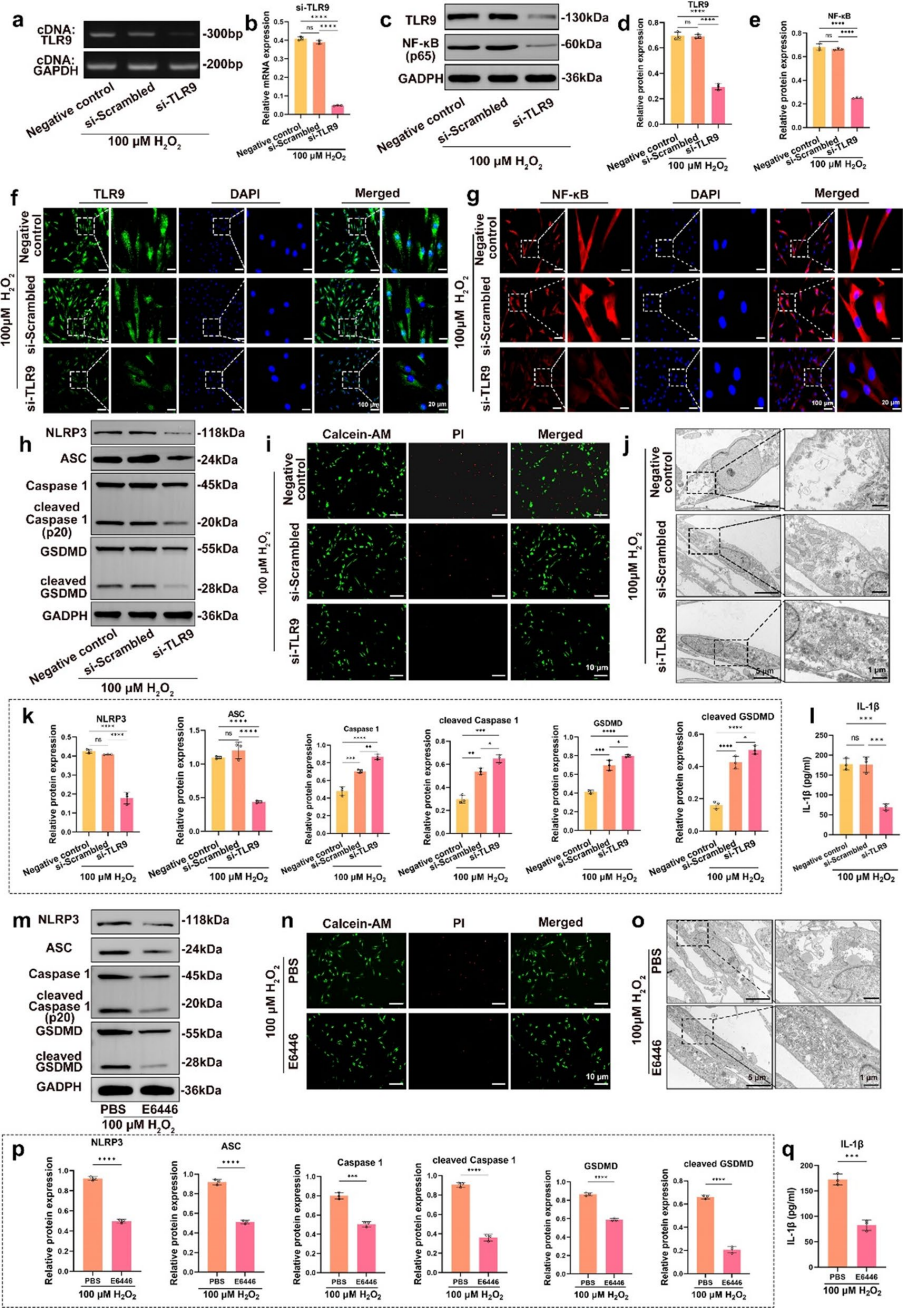
Knockdown or inhibition of TLR9 activation reduced NPC pyroptosis under OS. Knockdown by siRNA or inhibition of TLR9 activation by E6446 with 100 µM H_2_O_2_-treated the NPCs for 24 h. **a** Agar gelatin electrophoresis images of TLR9 cDNA in the negative control, si-Scrambled, and si-TLR9 groups. **b** RT-qPCR quantitative analysis of TLR9 cDNA after siRNA knockdown in the negative control, si-Scrambled, and si-TLR9 groups. **c** Western blotting images of the expression levels of TLR9 and NF-κB after siRNA knockdown in the negative control, si-Scrambled, and si-TLR9 groups. **d**, **e** Quantitative analysis of the protein level of TLR9 and NF-κB after siRNA knockdown in the negative control, si-Scrambled, and si-TLR9 groups. **f**, **g** Immunofluorescence images of TLR9 and NF-κB in the negative control, si-Scrambled, and si-TLR9 groups. **h** Western blotting images of NLRP3, ASC, pro caspase-1, cleaved caspase-1, GSDMD, and cleaved GSDMD in the negative control, si-Scrambled, and si-TLR9 groups. **i** Fluorescence images of Calcein AM/PI staining in the negative control, si-Scrambled, and si-TLR9 groups. **j** TEM images of the NPCs in the negative control, si-Scrambled, and si-TLR9 groups. **k** Quantitative analysis of the expression levels of NLRP3, ASC, pro caspase-1, cleaved caspase-1, GSDMD, and cleaved GSDMD in the negative control, si-Scrambled, and si-TLR9 groups. **l** Quantitative analysis of IL-1β by ELISA in the negative control, si-Scrambled, and si-TLR9 groups. **m** Western blotting images of NLRP3, ASC, pro caspase-1, cleaved caspase-1, GSDMD, and cleaved GSDMD in the E6446- or PBS-treated NPCs. **n** Fluorescence images of Calcein AM/PI staining in the E6446- or PBS-treated NPCs. **o** TEM images of the NPCs in the E6446- or PBS-treated NPCs. **p** Quantitative analysis of the expression levels of NLRP3, ASC, pro caspase-1, cleaved caspase-1, GSDMD, and cleaved GSDMD in the E6446- or PBS-treated NPCs. **q** Quantitative analysis of IL-1β by ELISA in the E6446- or PBS-treated NPCs. *p < 0.05, **p < 0.01, ***p < 0.001, ****p < 0.0001

Recent studies have shown that OS activates the TLR9-NF-κB axis and promotes inflammasome activation, leading to inflammatory damage [32-34]. To demonstrate that OS-induced TLR9-NF-κB-NLRP3 axis activation was the cause of NPC pyroptosis, we knocked down TLR9 by siRNA and subsequently treated the cells with 100 µM H_2_O_2_ for 24 h. The results showed that in the siRNA-TLR9 group, the TLR9 gene transcript level was substantially downregulated, indicating that the knockdown was effective (Fig. 3a, b). Furthermore, western blotting and immunofluorescence results showed that in the siRNA-TLR9 group, the levels of TLR9 and NF-κB proteins were substantially downregulated, which further indicated that the knockdown was effective (Fig. 3c–g and Additional file 2: Fig. S1b). Western blotting also showed that knockdown of TLR9 decreased H_2_O_2_-induced NLRP3 inflammasomes activation and the levels of pyroptosis-associated proteins (ASC, caspase-1, cleaved caspase-1, GSDMD, and cleaved GSDMD) and blocked NPC pyroptosis (Fig. 3h, k). In addition, ELISA results showed that TLR9 knockdown resulted in a substantial decrease in IL-1β expression (Fig. 3l). The viability of NPCs under OS substantially improved after TLR9 knockdown (Fig. 3i). TEM showed that TLR9 knockdown reduced H_2_O_2_-induced cell swelling (Fig. 3j).

In addition, we inhibited TLR9 activation with E6446 to verify NPC response under OS. The results showed that NLRP3 inflammasomes and pyroptosis-related proteins (ASC, caspase-1, cleaved caspase-1, GSDMD, and cleaved GSDMD) were downregulated in the E6446 group (Fig. 3m, p). Furthermore, ELISA showed that IL-1β expression was reduced after inhibition of TLR9 activation (Fig. 3q). Inhibition of TLR9 activation improved NPC viability under OS (Fig. 3n). TEM showed that the inhibition of TLR9 activation attenuated H_2_O_2_-induced cell swelling (Fig. 3o). In conclusion, these data suggest that knockdown or inhibition of TLR9 activation blocked NPC pyroptosis induced by TLR9-NF-κB-NLRP3 signaling.

### OS induced mPTP opening and cytosolic mtDNA over-release in NPCs

**Fig. 4:**
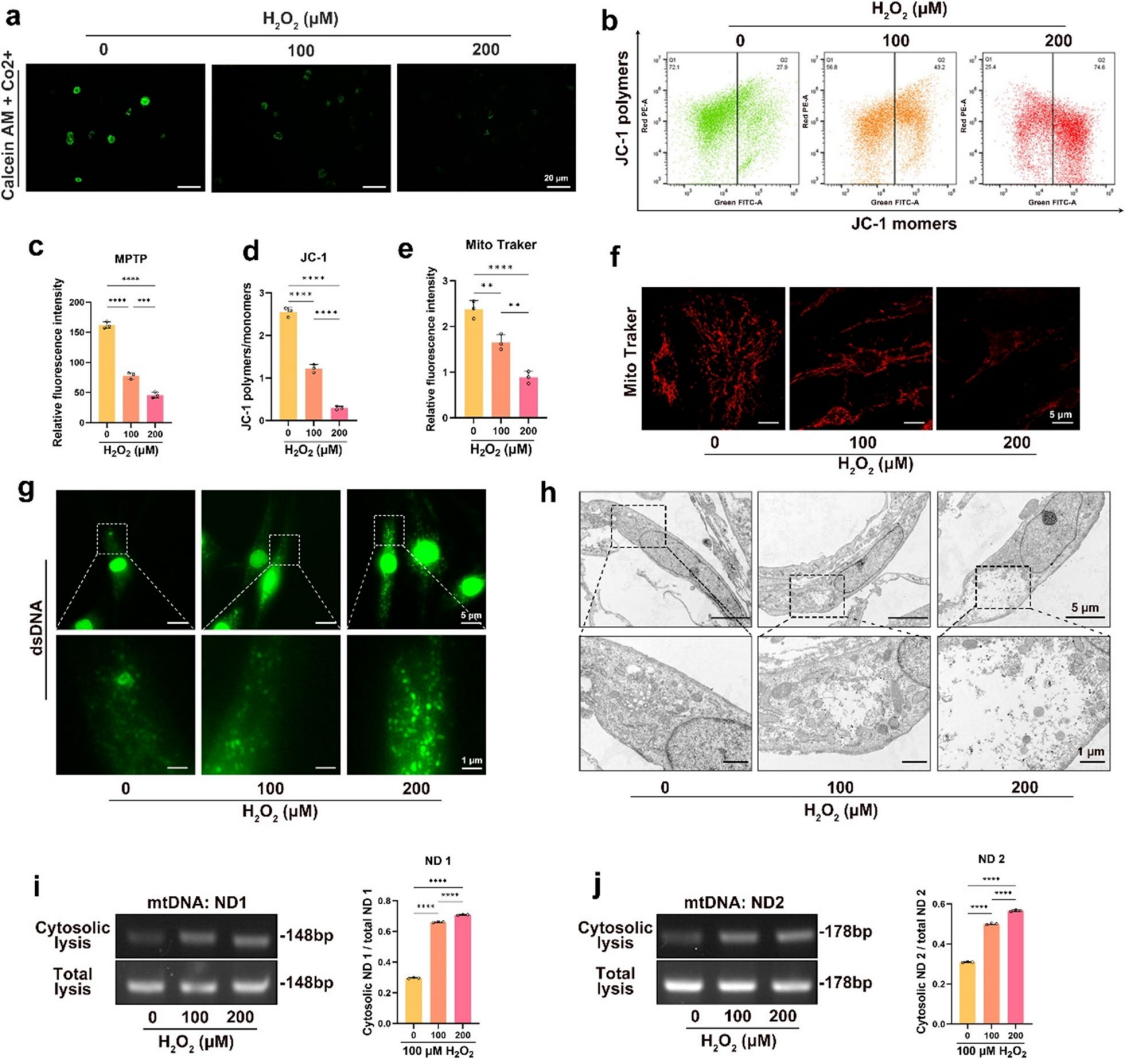
OS induced mPTP opening and cytosolic mtDNA over-release in NPCs. Human NPCs was cultured with 0, 100, and 200 µM H_2_O_2_ for 24 h. **a**, **c** Fluorescence images of Calcein AM/Co2^+^ staining and quantitative analysis of fluorescence intensity for the opening of mPTPs in the NPCs. **b**, **d** Scatter plots of JC-1 staining and quantitative analysis of MMP in NPCs. **e, f** Quantitative analysis of fluorescence intensity and fluorescence images of Mito-tracker for mitochondrial activity in the NPCs. **g** Fluorescence images of dsDNA (green) in the NPCs. **h** TEM images of the NPCs. **i**, **j** Agar gelatin electrophoresis images of mtDNA (ND1 and ND2) in the cytoplasmic or total lysates in NPCs. *p < 0.05, **p < 0.01, ***p < 0.001, ****p < 0.0001

After mitochondrial damage, mPTP opening and DAMPs are released into the cytosol, which can influence cell destiny [35]. OS can lead to an increase in mPTP opening, release of mtDNA into the cytosol, and activation of the TLR9-NF-κB axis [36]. To assess whether OS resulted in mPTP opening, we treated NPCs with different doses of H_2_O_2_, and the results showed that the fluorescence intensity of Calcein-AM in the mitochondria decreased with increasing doses of H_2_O_2_, which indicated an increase in mPTP opening (Fig. 4a, c). The voltage reduction of MMP can cause mPTP to open. Our data showed that the ratio of JC-1 polymers to monomers decreased in the H_2_O_2_-treated groups, indicating that OS increased the number of mPTP openings in the NPCs (Fig. 4b, d). Mitochondria-specific fluorescence staining of live cells showed a decrease in mitochondrial fluorescence intensity with increasing doses of H_2_O_2_, indicating increased mitochondrial damage caused by OS (Fig. 4e, f). In addition, immunofluorescence assays showed that mtDNA fluorescence intensity in the cytosol was enhanced with increasing H_2_O_2_ doses in NPCs (Fig. 4g and Additional file 2: Fig. S3a). TEM showed substantial swelling of the mitochondria in the H_2_O_2_-treated group, which is a characteristic of mPTP opening (Fig. 4h).

To further clarify whether OS leads to the abnormal release of mtDNA, we treated NPCs with different doses of H_2_O_2_, extracted cytosolic DNA, and visualized it by PCR. The results showed that in the H_2_O_2_-treated groups, the abundance of ND1 and ND2 (mtDNA gene fragment) were increased in the cytosol, while the total intracellular DNA abundance was not increased (Fig. 4i, j). These results indicate that OS leads to the opening of mPTP and over-release of cytosolic mtDNA, which activates the TLR9-NF-κB axis and NPC pyroptosis.

### Inhibition of mPTP opening blocked cytosolic mtDNA release and reduced NPC pyroptosis

**Fig. 5:**
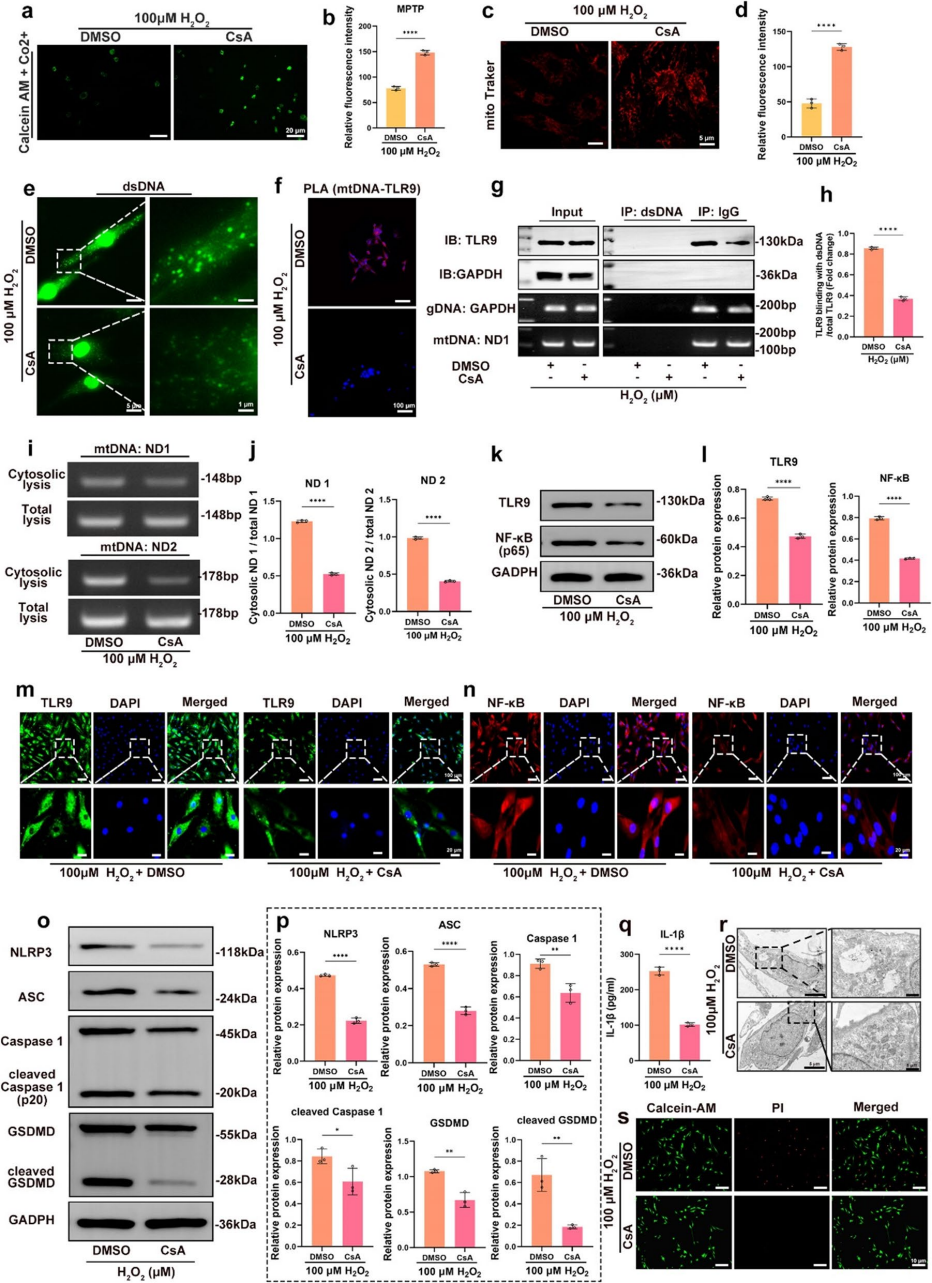
Inhibition of mPTP opening blocked cytosolic mtDNA release and reduced NPC pyroptosis. CsA or DMSO + 100 µM H_2_O_2_ was used to treat NPCs for 24 h. **a**, **b** Fluorescence images of Calcein AM/Co2^+^ staining and quantitative analysis of fluorescence intensity for the opening of mPTPs in the CsA or DMSO + 100 µM H_2_O_2_ treated NPCs. **c**, **d** Fluorescence images of Mito-tracker and quantitative analysis of fluorescence intensity for mitochondrial activity in the CsA- or DMSO-treated NPCs. **e** Fluorescence images of dsDNA (green) in the CsA or DMSO + 100 µM H_2_O_2_ treated NPCs. **f** The PLA immunofluorescence images of mtDNA-TLR9 interaction in the CsA or DMSO + 100 µM H_2_O_2_ treated NPCs. **g**, **h** Immunoprecipitation images of mtDNA-TLR9 interaction and quantitative analysis of the level of mtDNA-TLR9 interaction in the CsA or DMSO + 100 µM H_2_O_2_ treated NPCs. **i** Agar gelatin electrophoresis images of mtDNA (ND1 and ND2) in the cytoplasmic or total lysates in the CsA or DMSO + 100 µM H_2_O_2_ treated NPCs. **j** Quantitative analysis of the level of mtDNA (ND1 and ND2) in the cytoplasm in the CsA or DMSO + 100 µM H_2_O_2_ treated NPCs. **k**, **l** Western blotting images and quantitative analysis of the expression levels of TLR9 and NF-κB in the CsA- or DMSO-treated NPCs. **m**, **n** Immunofluorescence images of TLR9 and NF-κB in the CsA- or DMSO-treated NPCs. **o** Western blotting images of NLRP3, ASC, pro caspase-1, cleaved caspase − 1, GSDMD, and cleaved GSDMD in the CsA- or DMSO-treated NPCs. **p** Quantitative analysis of the expression levels of NLRP3, ASC, pro caspase-1, cleaved caspase-1, GSDMD, and cleaved GSDMD in the CsA- or DMSO-treated NPCs. **q** Quantitative analysis of IL-1β by ELISA in the CsA- or DMSO-treated NPCs. **r** TEM images of the NPCs in the CsA- or DMSO-treated. **s** Fluorescence images of Calcein AM/PI staining in the CsA- or DMSO-treated NPCs. *p < 0.05, **p < 0.01, ***p < 0.001, ****p < 0.0001

To further demonstrate that mPTP opening can lead to increased cytosolic mtDNA release, TLR9-NF-κB-NLRP3 axis activation, and NPC pyroptosis, NPCs were treated with CsA, a specialized inhibitor of mPTP opening. The results showed that in the CsA group, mPTP opening, mitochondrial damage, and release of mtDNA were reduced (Fig. 5a–e and Additional file 2: Fig. S3b). The PLA signal and immunoprecipitation results showed that in the CsA group, fluorescence signal intensity decreased (Fig. 5f and Additional file 2: Fig. S2b), and the level of mtDNA-TLR9 immunoprecipitation was reduced (Fig. 5g, h). These data suggest that inhibition of mPTP opening blocks mtDNA-TLR9 interaction. In addition, PCR and RT-qPCR results showed that in the CsA group, the level of cytosolic mtDNA was reduced (Fig. 5i, j). The western blotting results showed reduced levels of TLR9 and NF-κB protein in the CsA group (Fig. 5k, l). These data indicate that inhibition of mPTP opening can reduce mtDNA release and TLR9 and NF-κB activation. Furthermore, in the CsA group, the levels of the NLRP3 inflammasomes, ASC, caspase-1, cleaved caspase-1, GSDMD, cleaved GSDMD, and IL-1β were downregulated (Fig. 5o–q). TEM and fluorescence microscopy images showed that CsA alleviated swelling and pyroptosis, which demonstrated that inhibiting the opening of mPTP could reduce NPC pyroptosis (Fig. 5r, s). These data indicate that OS-mediated mPTP opening and mtDNA release contribute to TLR9-NF-κB-NLRP3 axis activation and pyroptosis in NPCs.

### Inhibiting the opening of mPTP and TLR9 activation can improve IVDD in vivo

**Fig. 6:**
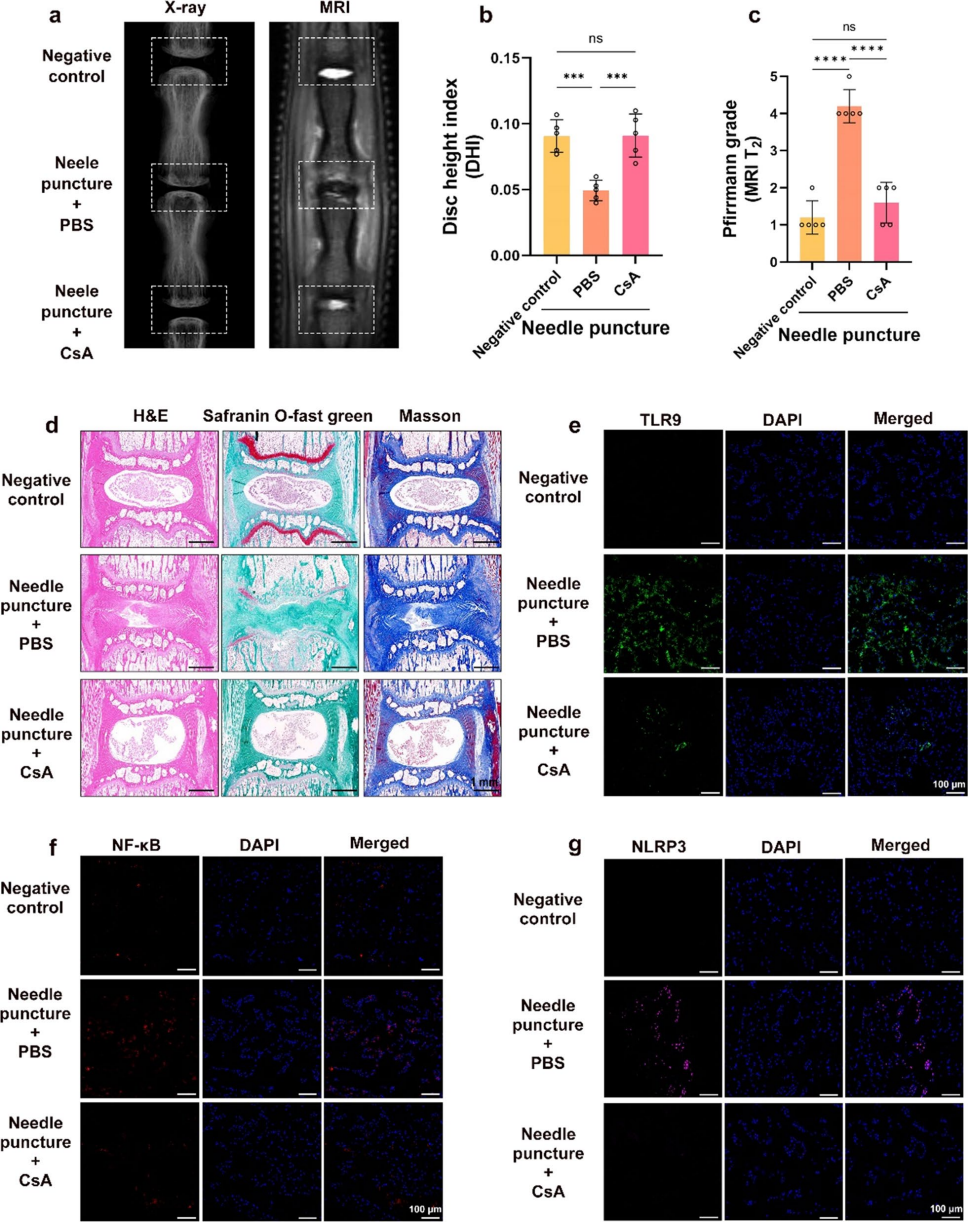
Inhibit the opening of mPTPs improved IVDD in vivo. A rat punctured IVDD model on Co7/8 and Co8/9 discs, and the Co8/9 disc was injected with CsA (10 µM), while Co7/8 disc was injected with the same amount of PBS. **a** X-ray and MRI T2 weighted images of rat tail. **b** The disc height index (DHI) was measured in the negative control, punctured + PBS injection, and punctured + CsA injection groups (n = 5). **c** The Pfirrmann grade was evaluated in the negative control, puncture + PBS injection, and puncture + CsA injection groups (n = 5). **d** Images of the H&E, safranin O-fast green, and Masson staining of rat discs in the negative control, punctured + PBS injection, and punctured + CsA injection groups. **e**–**g** Fluorescence images of TLR9, NF-κB, and NLRP3 in the negative control, punctured + PBS injection, and punctured + CsA injection groups. *p < 0.05, **p < 0.01, ***p < 0.001, ****p < 0.0001

**Fig. 7:**
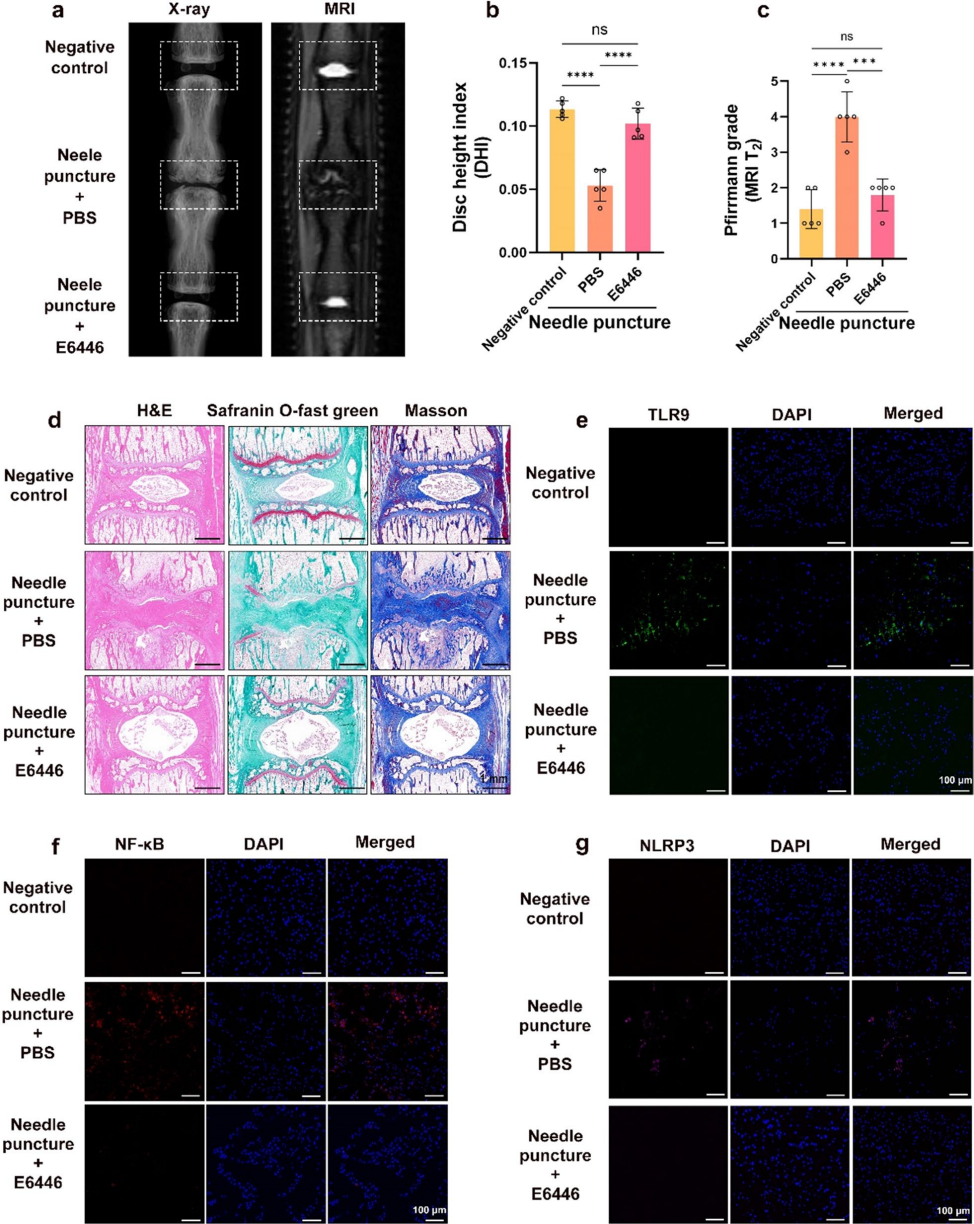
Inhibit TLR9 activation improved IVDD in vivo. A rat punctured IVDD model on Co7/8 and Co8/9 discs, and the punctured Co8/9 disc and injected with E6446 (10 µM), while punctured Co7/8 disc and injected with the same amount of PBS. **a** Representative X-ray and MRI T2 weighted images of a rat tail. **b** The disc height index (DHI) was measured in the negative control, puncture + PBS injection, and puncture + E6446 injection groups (n = 5). **c** The Pfirrmann grade was evaluated in the negative control, puncture + PBS injection, and puncture + E6446 injection groups (n = 5). **d** Representative images of the H&E, safranin O-fast green, and Masson staining of rat discs in the negative control, puncture + PBS injection, and puncture + E6446 injection groups. **e**–**g** Representative fluorescence images of TLR9, NF-κB, and NLRP3 in the negative control, puncture + PBS injection, and puncture + E6446 injection groups. *P < 0.05, **P < 0.01, ***P < 0.001, ****P < 0.0001

To assess the role of mPTP opening and TLR9 activation in IVDD, we used pharmacological interventions in a rat IVDD model. One month after pharmacological intervention, X-ray analysis showed a substantial reduction in disc height in the PBS injection group compared with normal discs, whereas the reduction in disc height in the CsA or E6446 injection group was not substantial (Figs. 6a, b and 7a, b). NP tissues in the PBS injection group were heterogeneous, and the T2-weighted signal was substantially lower than that in the CsA or E6446 injection groups (Figs. 6a, c and 7a, c). The histopathological results showed that in the PBS injection group, the NP was structurally disorganized, with increased fibrosis, and the boundaries between AF and NP could not be clearly distinguished. In contrast, the histological degeneration of the IVD was substantially lower in the CsA or E6446 injection groups than in the PBS injection group (Figs. 6d and 7d). Immunofluorescence results showed that, compared with the PBS injection group, the expression levels of TLR9, NF-κB, and NLRP3 were substantially lower in the CsA and E6446 injection groups (Figs. 6e–g and 7e–g). These data indicate that inhibiting the opening of mPTP and TLR9 activation can improve the degree of IVDD in the rat model.

## Discussion

**Fig. 8:**
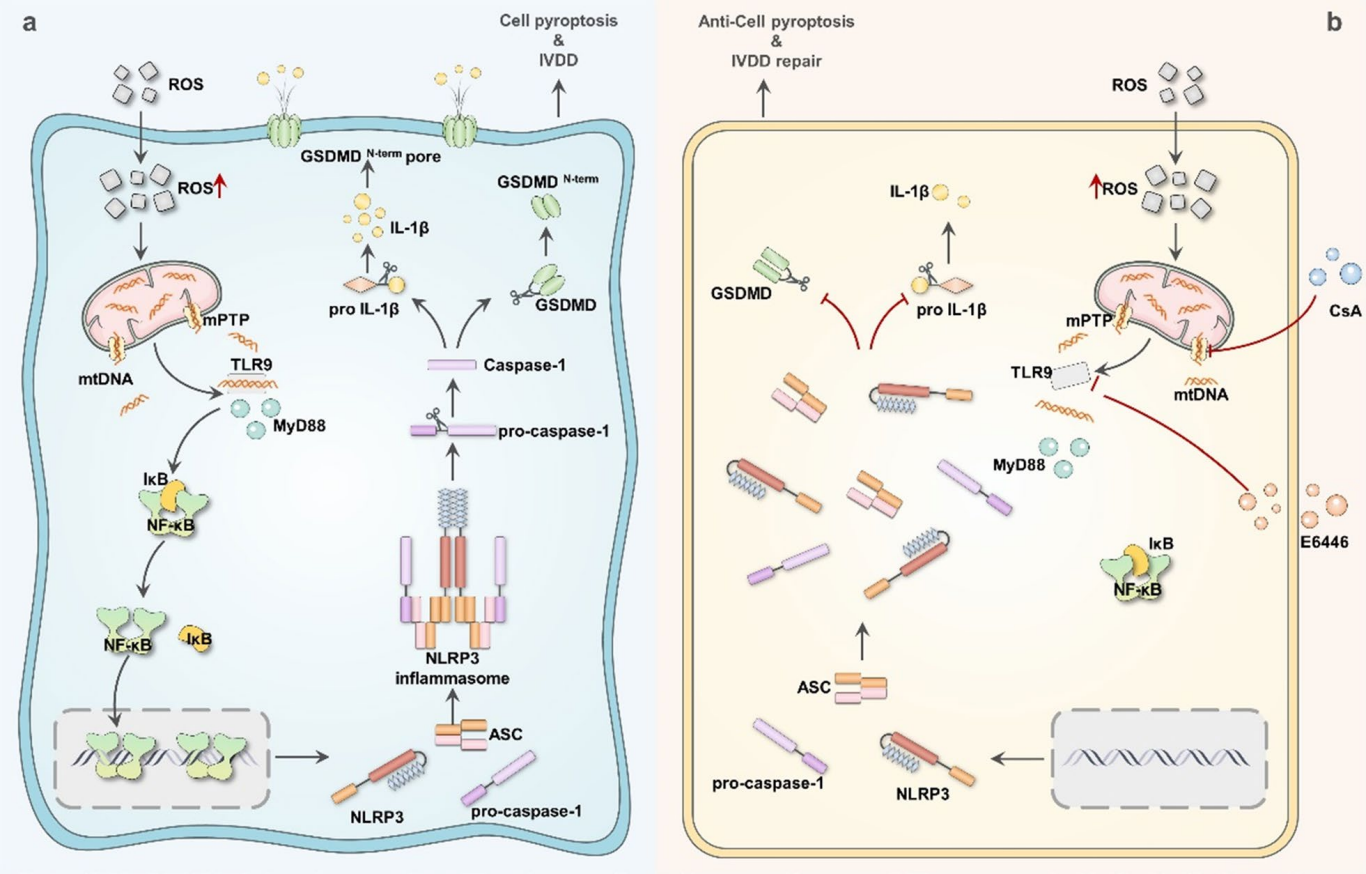
Mitochondrial DNA induces nucleus pulposus cell pyroptosis via the TLR9-NF-κB-NLRP3 axis. Graphical abstract of the critical roles of mtDNA via mPTP opening release into the cytoplasm and TLR9-NF-κB-NLRP3 axis-dependent NPCs pyroptosis in IVDD. **a** In IVDD, mtDNA via mPTP opening release into the cytoplasm triggered TLR9-NF-κB-NLRP3 axis-dependent NPCs pyroptosis. **b** The antagonist of mPTP opening (CsA) and TLR9 (E6446) mitigated NPCs pyroptosis and IVDD by inhibiting the TLR9-NF-κB-NLRP3 axis

Our study showed a correlation between the TLR9, NF-κB, and NLRP3 inflammasomes and IVDD. OS drives mtDNA-mediated TLR9-NF-κB-NLRP3 axis activation to induce pyroptosis in NPCs. Our study also demonstrated that the reason for mPTP opening and over-releasing mtDNA into the cytosol is because of OS-induced mitochondrial damage in NPCs. Furthermore, our study showed that TLR9 recognizes and binds mtDNA, which is a prerequisite for NF-κB-NLRP3 axis activation in NPCs. In addition, we demonstrated that TLR9 knockdown using siRNA substantially reduced NLRP3 inflammasomes activation and NPC pyroptosis in vitro. Furthermore, mechanistically, our study showed that pharmacological inhibition of mPTP opening by CsA blocked the release of mtDNA into the cytosol, and pharmacological inhibition of TLR9 activation by E6446 blocked TLR9 recognition and binding of mtDNA, both of which blocked the activation of the TLR9-NF-κB-NLRP3 axis and mitigated NPC pyroptosis in vitro and the development of IVDD in vivo (Fig. 8). In conclusion, our study suggests that mtDNA mediated the activation of TLR9-NF-κB-NLRP3 axis may be a potential biological marker for predicting the development of IVDD.

IVDD is accompanied by the following pathological changes: release of pro-inflammatory factors, inflammatory changes in the microenvironment, NPC injury and progressive loss, and extracellular matrix changes [3]. Our data showed a correlation between the levels of TLR9, NF-κB, and NLRP3 inflammasomes and the degree of IVDD. To understand IVDD, it is crucial to understand how NPCs detect and respond to damage. In numerous disorders, the TLR9-NF-B-NLRP3 axis activation can lead to the expression of multiple inflammatory factors. Our study confirmed that NPCs under OS could mediate the TLR9-NF-κB-NLRP3 axis activation and induce IL-1β expression and pyroptosis. Furthermore, our data showed that in NPCs under OS, TLR9 could recognize and bind mtDNA, which is critical for the activation of NF-κB and the NLRP3 inflammasomes. As DNA recognition receptors, cyclic GMP-AMP synthase (cGAS) and absent from melanoma 2 (AIM 2) also recognize DNA fragments [37]. The DNA recognition receptor cGAS preferentially recognizes long dsDNA, disordered DNA, or short dsDNA with unpaired open ends containing guanosine (y-type DNA), DNA-RNA hybrids, and exogenous DNA, such as viral DNA [38]. Although cGAS can recognize dsDNA, it is highly conserved in vertebrates [39]. AIM 2 is a DNA recognition mechanism complementary to TLR9 and cGAS. After binding to dsDNA in the cytosol, the conformation of AIM2 changes and polymerizes into ASC containing a CARD structure, inducing the expression of IL-1β [40, 41]. Our data do not rule out the potential role of cGAS and AIM 2 in the identification of self-DNA under OS. In short, it is still unclear which factors determine the activation of DNA receptors and the signal strength or duration of these factors when activating DNA receptors. Importantly, we described the critical role of TLR9 in recognizing mtDNA and mediating pyroptosis in NPCs.

The TLR9-NF-κB pathway is a key axis that recognizes and responds to mtDNA-induced DAMP activation. Nuevo et al. [36] showed that Opa1 deficiency in human muscle cells can lead to mitochondrial damage, mtDNA-mediated the TLR9-NF-κB axis activation, and the release of inflammatory factors, such as IL-1β. Consistent with these data, our study showed that OS drives mtDNA-mediated TLR9 activation and induces NPC pyroptosis. In addition, we found that TLR9 recognized and bound mtDNA, which is critical for NF-κB and the NLRP3 inflammasome activation. In this study, we examined whether mtDNA, as opposed to exogenous DNA, functions as a DAMP signal detected by TLR9 and regulates the NLRP3 inflammasomes activation and NPC pyroptosis, which is more compatible with the biological process of IVDD. We further clarified how OS leads to mtDNA release into the cytosol. Our data suggest that in NPCs, OS can result in mPTP opening and mtDNA release into the cytosol.

mPTP is a voltage-dependent anion channel. ROS induce mPTP opening, and once the mPTP is opened, calcium ions are translocated into the mitochondria, leading to MMP voltage changes and mitochondrial swelling, which eventually leads to mitochondrial lysis and mtDNA release into the cytosol [35]. mPTP opening can mediate mtDNA release and promote activation of inflammatory signaling pathways [42, 43]. When stress leads to mitochondrial damage, mtDNA induces the expression and release of cellular inflammatory factors, triggering cellular injury and even death [44]. Our study also demonstrated that the reason for mPTP opening and over-release of cytosolic mtDNA is because of OS-induced mitochondrial damage in NPCs. Moreover, our data suggest that OS resulted in the opening of mPTP and mtDNA release into the cytosol, which acted as a DAMP signal to activate the TLR9-NF-κB-NLRP3 axis, inducing NPC pyroptosis and IVDD. Although the function of mPTP is well-defined, the molecular components and regulatory mechanisms of mPTP remain unclear and require further study. In addition, previous studies reported that NRF2 can control cellular oxidation levels and oxidative signaling by regulating the expression of related genes that are involved in the programmed functional regulation of OS. Therefore, further studies on the role of NRF2 may provide new perspectives for intervention in IVDD [45, 46].

As a multi-protein complex, the NLRP3 inflammasome can respond to both exogenous and endogenous danger signals [14]. Once the NLRP3 inflammasome is activated, it activates GSDMD via caspase-1, releasing IL-1β and inducing inflammatory death (called pyroptosis) [47]. The NLRP3 inflammasome is associated with various disorders such as liver disease, cancer, and Alzheimer’s disease [34, 48, 49]. Shen et al. reported [50] that in a mouse model of diabetic nephropathy, activation of the TLR9-NF-κB axis mediated NLRP3 inflammasome expression, and knockdown TLR9 downregulated NLRP3 inflammasome expression and reduced inflammatory damage in mouse mesangial cells. In addition, previous studies have shown that the NLRP3 inflammasomes can induce NPC pyroptosis and is associated with IVDD [51-53]. Our data showed that OS induces NLRP3 inflammasome activation via the TLR9-NF-kB axis and mediates NPC pyroptosis and IVDD. Therefore, elucidating the connection between the NLRP3 inflammasome and pyroptosis will not only improve our knowledge of inflammatory damage, but also lead to novel therapeutic targets and approaches for IVDD associated with NLRP3 inflammasome activation.

This study still has the following shortcomings, first, the animal model of IVDD is unable to completely simulate human IVDD. Second, there is artificial trauma during the construction of the puncture IVDD model. There are several animal models of IVDD, but there is no ideal model so far, mainly because none of them can completely simulate the biomechanical environment of human IVDD. In addition, exploring new drug delivery systems can help in the prevention and treatment of IVDD. Therefore, the establishment of animal models and drug delivery systems that are more in accordance with the biological process of human IVDD can help to study the mechanism and treatment of IVDD.

## Conclusions

Our study suggested that the mtDNA-triggered TLR9-NF-B-NLRP3 axis induces NPC pyroptosis and IVDD development. Furthermore, we demonstrated that OS induced the opening of mPTP and mtDNA release could activate the TLR9-NF-κB-NLRP3 axis in NPCs. Our data also showed that inhibiting either mPTP from opening or TLR9 activation could mitigate NPC pyroptosis and IVDD development. Therefore, the mtDNA-TLR9-NF-κB-NLRP3 axis could serve as both a potential biomarker for IVDD and as a promising biological pathway for treating IVDD.

## Supplementary Information

**Additional file 1: Table S1.** Information of volunteers. **Table S2.** Information for antibodies. **Table S3.** Table of materials for western blotting. **Table S4.** Primers used in this study. **Table S5.** The parameters of radiography images. **Table S6.** Information of assays/instruments/software used in this study.

**Additional file 2: Figure S1.** The immunofluorescence images and quantitative analysis of fluorescence intensity of TLR9 and NF-κB. **Figure S2.** The immunofluorescence images and quantitative analysis of fluorescence intensity of PLA (mtDNA-TLR9). **Figure S3.** The immunofluorescence images and quantitative analysis of fluorescence intensity of dsDNA.

## Data Availability

The datasets during the current study are available from the corresponding author on reasonable request.

## Abbreviations

NPC: Nucleus pulposus cell
IVDD: Intervertebral disc degeneration
mtDNA: Mitochondrial DNA
mPTP: Mitochondrial permeability transition pores
LBP: Low back pain
ECM: Extracellular matrix
OS: Oxidative stress
MMP: Mitochondrial membrane potential
DAMP: Damage-associated molecular pattern
ASC: Apoptosis-associated speck-like protein
GSDMD: Gasdermin D
CsA: Cyclosporine
H&E: Hematoxylin and eosin
siRNA: Small-interfering RNA
dsDNA: Double-stranded DNA
PLA: Proximity ligation assay
TEM: Transmission electron microscopy
