# Supplementary material for: Mitochondrial DNA induces nucleus pulposus cell pyroptosis via the TLR9-NF-κB-NLRP3 axis
Source: J Transl Med. 2023 Jun 15;21:389. doi: 10.1186/s12967-023-04266-5 (PMC10273761; doi:10.1186/s12967-023-04266-5)
Supplement: Supplementary file 1 — Additional file 1: Table S1. Information of volunteers. Table S2. Information for antibodies. Table S3. Table of materials for western blotting. Table S4. Primers used in this study. Table S5. The parameters of radiography images. Table S6. Information of assays/instruments/software used in this study. [file 12967_2023_4266_MOESM1_ESM.docx]

Table S1. Information of volunteers

| **Number** | **Gender** | **Age (years)** | **Diagnosis** | **Segment** | **Pfirrmann grade** | **Operation Method** |
| --- | --- | --- | --- | --- | --- | --- |
| Case 1 | Male | 23 | Idiopathic scoliosis | T12-L1 | Ⅰ | Open operation |
| Case 2 | Female | 35 | Lumbar disc herniation | L4-L5 | Ⅱ | Endoscopic excision disc |
| Case 3 | Female | 46 | Lumbar disc herniation | L4-L5 | Ⅲ | Endoscopic excision disc |
| Case 4 | Female | 22 | Spinal trauma | T12-L1 | Ⅰ | Open operation |
| Case 5 | Female | 35 | Lumbar disc herniation | L5-S1 | Ⅳ | Endoscopic excision disc |
| Case 6 | Male | 28 | Lumbar disc herniation | L4-L5 | Ⅱ | Endoscopic excision disc |
| Case 7 | Female | 55 | Lumbar disc herniation | L4-L5 | Ⅳ | Open operation |
| Case 8 | Female | 41 | Lumbar disc herniation | L5-S1 | Ⅲ | Endoscopic excision disc |
| Case 9 | Male | 27 | Idiopathic scoliosis | T11-T12 | Ⅰ | Open operation |
| Case 10 | Male | 40 | Lumbar disc herniation | L2-L3 | Ⅲ | Endoscopic excision disc |
| Case 11 | Male | 42 | Lumbar disc herniation | L4-L5 | Ⅱ | Endoscopic excision disc |
| Case 12 | Male | 40 | Lumbar disc herniation | L3-L4 | Ⅳ | Endoscopic excision disc |
| Case 13 | Male | 37 | Spinal trauma | L1-L2 | Ⅰ | Open operation |
| Case 14 | Female | 23 | Lumbar disc herniation | L4-L5 | Ⅲ | Endoscopic excision disc |
| Case 15 | Male | 17 | Idiopathic scoliosis | L1-L2 | Ⅰ | Open operation |
| Case 16 | Female | 20 | Lumbar disc herniation | L4-L5 | Ⅱ | Endoscopic excision disc |
| Case 17 | Male | 37 | Lumbar disc herniation | L4-L5 | Ⅳ | Endoscopic excision disc |
| Case 18 | Male | 28 | Lumbar disc herniation | L5-S1 | Ⅲ | Endoscopic excision disc |
| Case 19 | Female | 22 | Lumbar disc herniation | L5-S1 | Ⅱ | Endoscopic excision disc |
| Case 20 | Male | 65 | Lumbar disc herniation | L4-L5 | Ⅳ | Open operation |

Table S2. Information for antibodies

| **Antibodies** | **Source** | **Catalog** | **Dilution ratio** |
| --- | --- | --- | --- |
| anti-TLR9 | abcam | ab134368 | 1:3000 |
| anti-NF-κB | abcam | ab207297 | 1:2000 |
| anti-NLRP3 | abcam | ab263899 | 1:3000 |
| anti-ASC | abcam | ab150368 | 1:3000 |
| anti-caspase-1 | Cell Signaling Technology | 3866S | 1:3000 |
| anti-cleaved caspase-1 | Cell Signaling Technology | 4199S | 1:3000 |
| anti-GSDMD | Cell Signaling Technology | 97558S | 1:3000 |
| anti-cleaved GSDMD | Cell Signaling Technology | 37349 | 1:3000 |
| anti-GAPDH | abcam | ab8245 | 1:2000 |
| Goat Anti-Rabbit IgG H&L (HRP) | abcam | ab205718 | 1:5000 |
| Goat Anti-Mouse IgG H&L (HRP) | abcam | ab97023 | 1:5000 |

Table S3. Table of materials for western blotting

| **Reagent** | **Source** | **Catalog** |
| --- | --- | --- |
| Polyvinylidene fluoride membrane (PVDF) | Servicebio | G6015 |
| RIPA Buffer | Servicebio | G2002 |
| SDS-PAGE Sample Prep Kit | Pierce | 89888 |
| Phosphatase inhibitor | Servicebio | G2007 |

Table S4. Primers used in this study

| **Gene Name** | **Forward Primer** | **Reverse Primer** |
| --- | --- | --- |
| si-TLR9 | 5'-AGCTTAACCTGTCCTTCAATTAC-3' | 5'-AAUUGAAGGACAGGUUAAGCU-3' |
| si-Scrambled | 5'-CUUAACCUGUCCUUCAAUUAC-3' | 5'-CUGCAAAUACUAGAUGUAAGC-3' |
| Human-TLR9 | 5'-ACTGGCTGTTCCTGAAGTCTGTG-3' | 5'-CAGGTTTAGCTCTTCCAGGGTG-3' |
| Human-GAPDH | 5'-GGAAGCTTGTCATCAATGGAAATC-3' | 5'-TGATGACCCTTTTGGCTCCC-3' |
| Human-MT-ND1 | 5'-TCCTAATGCTTACCGAACGAAA-3' | 5'-ATGGTAGATGTGGCGGGTTT-3' |
| Human-MT-ND2 | 5'-GTATTTCCTCACGCAAGCAACC-3' | 5'-CTCTGGGACTCAGAAGTGAAAGG-3' |

Table S5. The parameters of radiography images

| **X-ray (UltraFocus DXA, Faxitron)** | | | |
| --- | --- | --- | --- |
| Exposure | 50 mAs | Penetration power | 48 kV |
| **MRI (BioSpec70/20USR, Bruker)** | | | |
| Time-to-repetition | 3000 | Flip angle | 90 |
| Time-to-echo | 40 | Field of view | 60×20 |
| Number of Excitation | 5 | Matrix | 300×100 |

Table S6. Information of assays/instruments/software used in this study

| **Methods** | **Critical commercial kit/Instruments** | **Source** | **Catalog** |
| --- | --- | --- | --- |
| Tyramide signal  amplification immunofluorescence | Cy3 TSA Fluorescence System Kit | ApexBio | K1051 |
| siRNA | Lipofectamine 8000 Kit | Beyotime | C0533 |
| RNA reverse transcription | SweScript RT I First Strand cDNA Synthesis Kit | Servicebio | G3330 |
| General PCR | 2×Fast Pfus PCR Master Mix | Servicebio | G3305 |
| RT-PCR | 2 × SYBR Green qPCR Master Mix (None ROX) | Servicebio | G3320 |
| Cytoplasmic ROS | H2DCFDA | Invitrogen | D399 |
| Mitochondrial ROS | MitoSOX™ Red Mitochondrial Superoxide Indicator Kit | Invitrogen | M36008 |
| Mitochondrial membrane potential | MitoProbe™ JC-1 Assay Kit | Invitrogen | M34152 |
| Mitochondrial quality test | Mito-Tracker Red CMXRos Assay Kit | Invitrogen | M7512 |
| mPTP | Mitochondrial Permeability Transition Pore Assay Kit | Beyotime | C2009S |
| NPC viability assay | Calcein AM/PI Kit | Beyotime | C2015S |
| human IL-1β | IL-1β humanELISA Kit | Invitrogen | KAC1211 |
| Cytoplasm DNA and total DNA extraction | NE-PER^TM^ Nuclear and Cytoplasmic Extraction Reagents kit | Thermo Scientific | 78833 |
| Proximity ligation assy | Duolink^®^ In situ PLA^®^ Kit | SigmaAldrich | DUO94104 |
| Flow cytometer | FACSCablibur flow cytometer | BD Biosciences |  |
| Microscope | Eclipse 80i | Nikon |  |
| Transmission electron microscope | HT7800 | Hitachi |  |
