# Supplementary material for: Mitochondrial DNA induces nucleus pulposus cell pyroptosis via the TLR9-NF-κB-NLRP3 axis
Source: J Transl Med. 2023 Jun 15;21:389. doi: 10.1186/s12967-023-04266-5 (PMC10273761; doi:10.1186/s12967-023-04266-5)
Supplement: Supplementary file 2 — Additional file 2: Figure S1. The immunofluorescence images and quantitative analysis of fluorescence intensity of TLR9 and NF-κB. Figure S2. The immunofluorescence images and quantitative analysis of fluorescence intensity of PLA (mtDNA-TLR9). Figure S3. The immunofluorescence images and quantitative analysis of fluorescence intensity of dsDNA. [file 12967_2023_4266_MOESM2_ESM.docx]

**Fig. S1 The immunofluorescence images and quantitative analysis of fluorescence intensity of TLR9 and NF-κB**

**
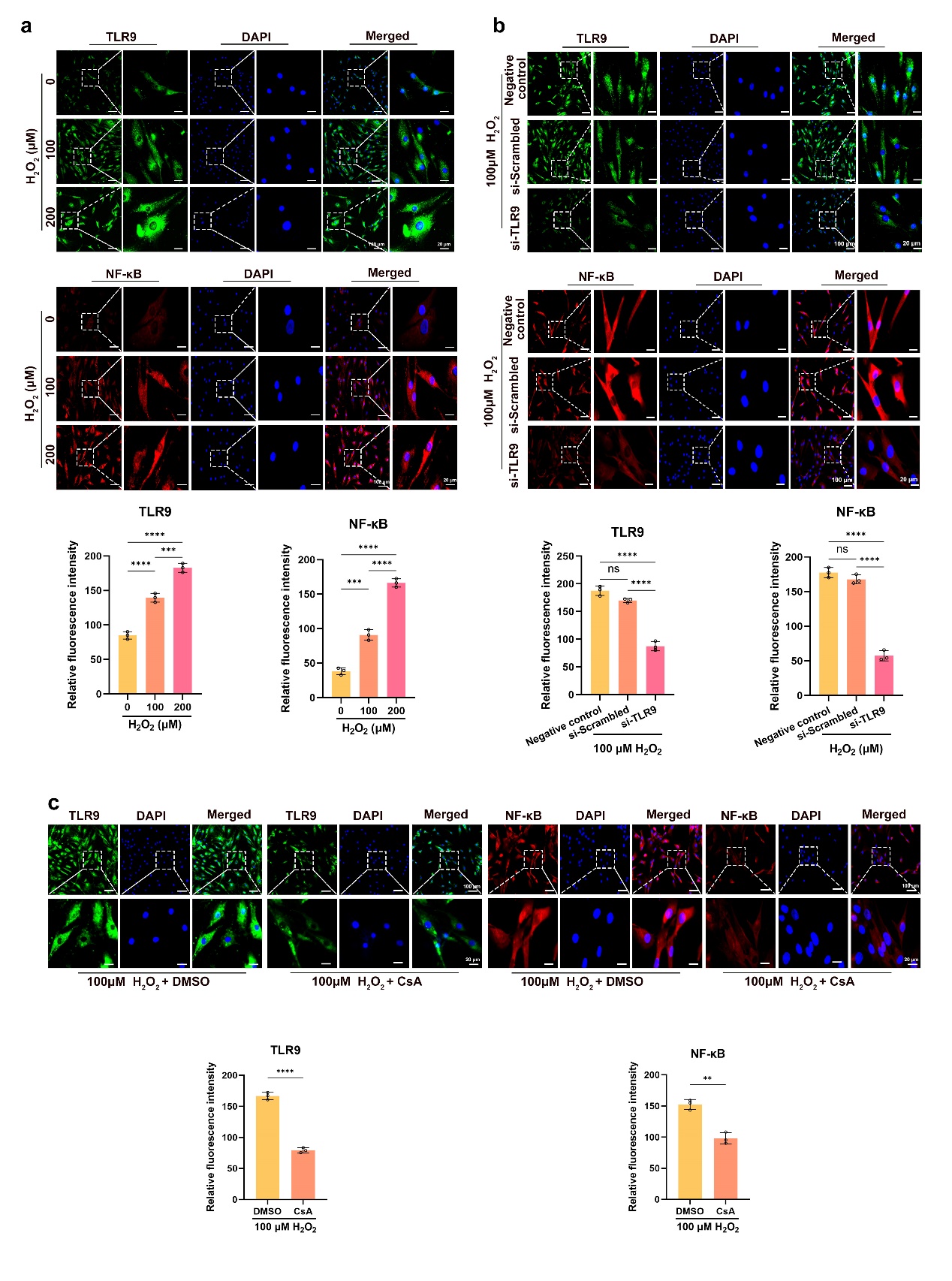
**

a. Immunofluorescence images and quantitative analysis of fluorescence intensity of TLR9 and NF-κB in the H_2_O_2_-treated NPCs

b. Immunofluorescence images and quantitative analysis of fluorescence intensity of TLR9 and NF-κB after siRNA knockdown.

c. Immunofluorescence images and quantitative analysis of fluorescence intensity of TLR9 and NF-κB in CsA or DMSO-treated NPCs after exposed 100 μm.

*P < 0.05, **P < 0.01, ***P < 0.001, ****P < 0.0001.

**Fig. S2 The immunofluorescence images and quantitative analysis of fluorescence intensity of PLA (mtDNA-TLR9)**

**
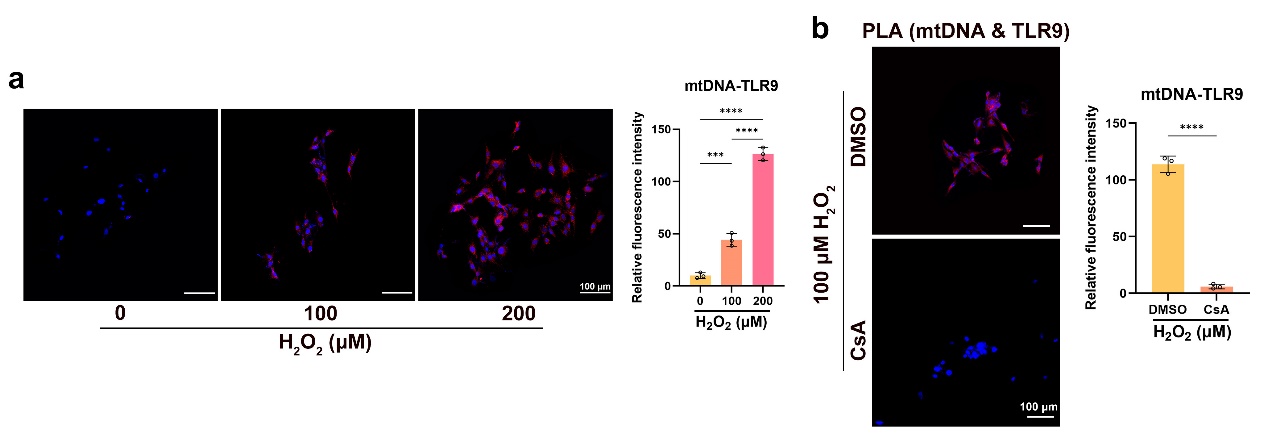
**

a. Immunofluorescence images and quantitative analysis of fluorescence intensity of PLA (mtDNA-TLR9) in the H_2_O_2_-treated NPCs

b. Immunofluorescence images and quantitative analysis of fluorescence intensity of PLA (mtDNA-TLR9) in CsA or DMSO-treated NPCs after exposed 100 μm.

*P < 0.05, **P < 0.01, ***P < 0.001, ****P < 0.0001.

**Fig. S3 The immunofluorescence images and quantitative analysis of fluorescence intensity of dsDNA**

**
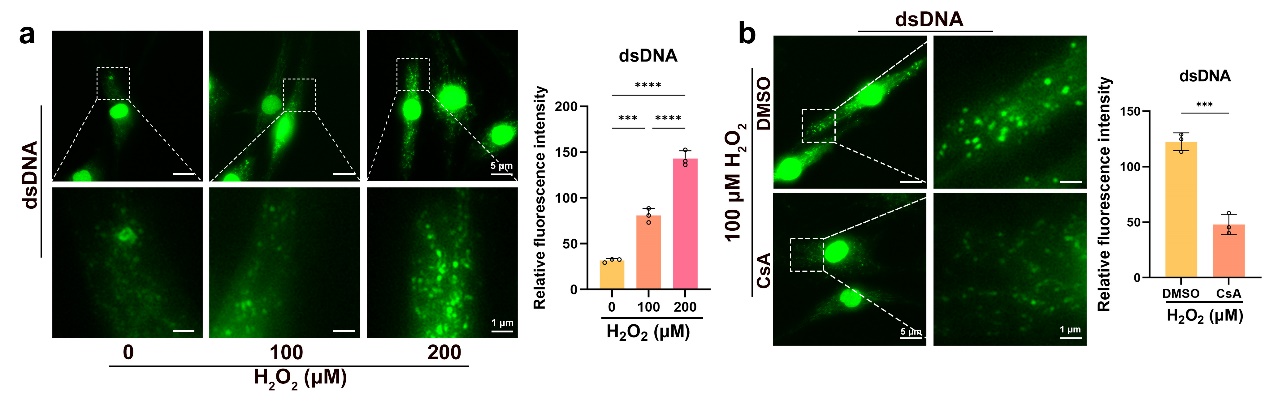
**

a. Immunofluorescence images and quantitative analysis of fluorescence intensity of dsDNA in the H_2_O_2_-treated NPCs

b. Immunofluorescence images and quantitative analysis of fluorescence intensity of dsDNA in CsA or DMSO-treated NPCs after exposed 100 μM H_2_O_2_.

*P < 0.05, **P < 0.01, ***P < 0.001, ****P < 0.0001.
